# Bioactives and Technological Quality of Functional Biscuits Containing Flour and Liquid Extracts from Broccoli By-Products

**DOI:** 10.3390/antiox12122115

**Published:** 2023-12-14

**Authors:** Benedetta Fanesi, Lama Ismaiel, Ancuta Nartea, Oghenetega Lois Orhotohwo, Anastasiya Kuhalskaya, Deborah Pacetti, Paolo Lucci, Pasquale Massimiliano Falcone

**Affiliations:** Department of Agricultural, Food and Environmental Sciences, Università Politecnica delle Marche, 60131 Ancona, Italy; b.fanesi@univpm.it (B.F.); l.ismaiel@pm.univpm.it (L.I.); a.nartea@univpm.it (A.N.); o.orhotohwo@pm.univpm.it (O.L.O.); a.kuchalskaja@univpm.it (A.K.); d.pacetti@univpm.it (D.P.); p.m.falcone@univpm.it (P.M.F.)

**Keywords:** glucosinolates, vitamin A, vitamin E, polyphenols, bakery formulation, functional food, rheological properties, *Brassica* species

## Abstract

Broccoli by-products are an important source of health-promoting bioactive compounds, although they are generally underutilized. This study aimed to valorize non-compliant broccoli florets by transforming them into functional ingredients for biscuit formulation. A broccoli flour and three water/ethanol extracts (100:0, 75:25, 50:50; *v*/*v*) were obtained. The rheological properties and the content of bioactive compounds of the functional ingredients and biscuits were evaluated. The 50:50 hydroalcoholic extract was the richest in glucosinolates (9749 µg·g^−1^ DW); however, the addition of a small amount strongly affected dough workability. The enrichment with 10% broccoli flour resulted the best formulation in terms of workability and color compared to the other enriched biscuits. The food matrix also contributed to protecting bioactive compounds from thermal degradation, leading to the highest total glucosinolate (33 µg·g^−1^ DW), carotenoid (46 µg·g^−1^ DW), and phenol (1.9 mg GAE·g^−1^ DW) contents being present in the final biscuit. Therefore, broccoli flour is a promising ingredient for innovative healthy bakery goods. Hydroalcoholic extracts could be valuable ingredients for liquid or semi-solid food formulation.

## 1. Introduction

Oxidative stress and the modern lifestyle are the main influencing factors in human health problems. A variety of negative effects on body functionality can be triggered by the inhibition of our body’s natural antioxidant system’s ability to destroy free radicals [1]. Including more sources of antioxidants in our daily diets prevents such consequences. Natural antioxidants are essential secondary metabolites characterized by powerful antioxidant activity [2]. Pigments derived from various fruits and vegetables such as tocopherols [3,4,5], carotenoids [3,6], glucosinolates [7,8], and polyphenols [9,10] have been addressed in different studies in recent decades because of their multiple benefits for human health. Promising opportunities for their application in the food, pharmaceutical, beauty, and various other sectors have been recognized [2]. Carotenoids play a significant role as natural antioxidants and serve as precursors to vitamin A [11]. Tocopherols have demonstrated their ability to provide a defense against inflammatory conditions and cancer [12]. Glucosinolates and their isothiocyanates play a notable role in preventing and treating multiple chronic diseases such as cardiometabolic disorders [8]. Phenolic compounds also exert antioxidant, anti-inflammatory and anticancer activities [13].

Cruciferous vegetables including cauliflower, cabbage, brussels sprouts, and broccoli are known for their bioactive compounds which enhance the prevention of colorectal cancer and reduce the prevalence of numerous diseases [14]. The production of broccoli had doubled by 2019 compared to the late 19th century, and Italy represents the fourth largest producer after India, the USA, and Spain [15]. Broccoli is appreciated especially for its abundance of glucosinolates, which have been recognized as potential candidates for chronic disease prevention [8]. Twelve glucosinolates have been identified in eighty genotypes of broccoli florets, including glucobrassicin, 4-methoxyglucobrassicin, 4-hydroxyglucobrassicin, glucoerucin neoglucobrassicin and glucoraphanin [16]. Broccoli contains exceptionally high levels of bioactive components such as tocopherols, estimated at around 155–1.57 μg·g^−1^ dry weight (DW) in the leaves and florets, respectively [17]. In addition, broccoli had the highest tocopherol content compared to other *Brassicaceae* vegetables [3]. Lutein, β-carotene, violaxanthin, and neoxanthin were the predominant carotenoids in broccoli [3,17].

Unfortunately, 85–90% of broccoli’s total aerial biomass, including the leaves, stems, and non-compliant florets, is usually disposed of as by-products and underutilized [13,17,18]. These by-products contain high amounts of valuable nutrients that can be recovered and utilized, including vitamins, glucosinolates, carotenoids and chlorophylls. The evaluation of phytonutrients in broccoli by-products revealed that the leaf tissue contained higher levels of essential nutrients, such as β-carotene, vitamins E and K, minerals like Mn and Ca, as well as a greater total phenolic content (TPC) and DPPH antioxidant activity compared to the floral parts [17]. Various beneficial effects of broccoli by-products, such as antiobesity, antioxidation, anti-inflammation, anticancer and antifungal effects, have been confirmed in different studies [17,19,20]. An extract from broccoli sprouts enhanced the metabolism in individuals with obesity and poorly managed diabetes [8]. Broccoli leaf powder was regarded as a compelling and innovative ingredient suitable for gluten-free cake recipes [21]. Hence, broccoli by-products emerge as promising functional ingredients for incorporation into food products to valorize their health-promoting attributes.

Recently, increased interest has been shown towards new functional foods due to the beneficial health properties associated with their consumption. Further concerns have been raised regarding the use of food by-products as functional foods aimed at reducing the environmental impact of food production since the introduction of the circular economy principles to food science [22]. Different forms of broccoli waste have been used for the formulation of new functional food products with enhanced health benefits, for example snack bars [23], crackers [10], cakes [21] and bread [24]. The enhanced technological and functional attributes of these products have been acknowledged. Color is one of the most important parameters able to drive consumer choice [25]. The pigments present in broccoli (carotenoids and chlorophylls) provide an attractive green color when broccoli flour is employed in food formulations. For instance, its incorporation in cakes improved the attractiveness of gluten-free sponge cakes [21]. The use of broccoli flour in food formulations also has an effect on the texture of the final products. For instance, increasing the amount of broccoli flour led to an increase in the hardness of broccoli-containing crackers [10] and sponge cakes [21]. Most of the organoleptic attributes, including the flavor, appearance, crunchiness, and consistency, of bar snacks prepared with a broccoli–soybean–mangrove flour were favored by the vast majority of semi-trained panelists [23]. In this respect, it is important to valorize broccoli and its by-product ingredients.

In this work, we evaluated the enrichment of biscuits, one of the most popular bakery goods, using different functional ingredients from broccoli by-products. For this purpose, four ingredients (three liquid extracts and one flour) were obtained using eco-friendly (food-grade) solvents and cost-effective techniques. Therefore, we investigated the effect of their inclusion on the rheological properties of the doughs (i.e., extensional viscosity, spread and stickiness, color) as well as the final biscuits (i.e., texture profile, fracture, color). At the same time, the broccoli-derived ingredients and biscuits were analyzed to assess the content of bioactive compounds (i.e., glucosinolates, polyphenols, carotenoids, and tocopherols).

## 2. Materials and Methods

### 2.1. Broccoli By-Products and Derived Ingredients

Blanched frozen broccoli by-products were provided by a local company (O.R.T.O. Verde S.c.a.p.a., Senigallia, Italy). Broccoli by-products, accounting for non-compliant florets and stems, were stored at −40 °C before being freeze-dried (Superco engineering, CryoDryer 5, Augsburg, Germany). Freeze-drying was carried out using an automatic process consisting of a primary drying performed at 0.38 mbar and a secondary drying at 0.25 mbar. Dried broccoli was ground into flour (BF), and some of this flour was employed to make the liquid extracts. According to the Directive 2009/32/EC [26], the extraction solvents chosen to produce food ingredients were water and ethanol, which were employed in three different ratios (i.e., 100:0, 75:25, 50:50, *v*/*v*), hereafter referred to as 100W, 75W25ET, and 50W50ET, respectively. A single batch (50 mL) for each extract was prepared using a 1:15 (*w*/*v*) sample to solvent ratio, warmed at 40 °C for 1 h under stirring, as suggested by Bojorquez-Rodríguez et al. [27]. Later, they were centrifuged (Remi Elektrotechnik Ltd., Neya 16R, Mumbai, India) at 3500 rpm for 5 min at 4 °C to remove the residue and stored at −20 °C prior to chemical analysis and biscuit preparation.

### 2.2. Biscuits Preparation

**Figure 1:**
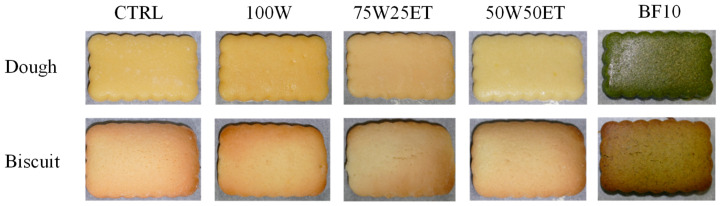
Control, enriched doughs and their biscuits prepared with broccoli-derived ingredients.

BF and extracts were added as functional ingredients in the biscuit formulations, for a total of 4 doughs. In detail, 10% BF was used to substitute wheat flour, while liquid extracts (30 mL each) replaced some of the sunflower oil. A control dough was also prepared. The ingredients used for the preparation of biscuits are listed in Table 1 and the samples are shown in Figure 1.

A planetary mixer (Kenwood, Model KWL90.244SI, Woking, UK) was used to mix the ingredients and prepare the dough. At first, eggs, sugar and cream of tartar were mixed at maximum speed (200 rpm) for 7 min. The liquid ingredients, i.e., sunflower oil, milk, and broccoli extracts, were poured and mixed for another 3 min. After this, flour, broccoli flour, salt, and sodium bicarbonate were slowly added, and the mixing speed was reduced to about 70 rpm for 4 min. The dough was left to rest at a refrigerated temperature (4 °C) for 30 min. The dough was rolled on a flat surface with a rolling pin set at a constant rolling thickness of 1 cm. Half of the dough was employed for rheological measurements, and half was sized with a biscuit mold (50 × 30 mm). For each dough, 24 biscuits were obtained and baked in a ventilated oven (Bosch, HSG636ES1, Munich, Germany) at 180 °C for 18 min and then cooled to room temperature for 30 min.

### 2.3. Rheological and Mechanical Analysis

Rheological and mechanical analysis of dough and biscuit samples was performed using a universal testing machine (Zwick Roel, model 1 KN, Ulm, Germany) equipped with specific tools.

#### 2.3.1. Lubricated Biaxial Extension of Doughs

Dough workability was first investigated by evaluating the strain resistance under large deformations (up to 75%) preceding and exceeding the fracture point. Two cylindrical plexiglass probes with a height of 11 mm and a diameter of 15 mm and with lubricated bottom and upper surfaces were used. The load (N) was registered as a function of time (s) and load-line displacement (mm), while the load-line displacement rate was set at 50 mm·min^−1^ (0.00083 m·s^−1^).

The apparent biaxial extensional viscosity (ABEV) was calculated:

ABEV = (2 × F_(t)_ × h_(t)_)/(πr^2^ × v)

where F_(t)_ is the load (N) at time t, h_(t)_ is the height (m) and r is the radius (m) of the dough specimen at time t, and v is the load-line displacement speed (m·s^−1^).

The hardening rate of the dough, expressed as the maximum increase in ABEV as a function of strain rate, was calculated in the range of strain rate preceding the fracture point. The latter was expressed as the strain rate (s^−1^) where the ABEV reached its maximum value. The lubricated biaxial extension test was replicated 10 times for each dough formulation.

#### 2.3.2. Spread and Stickiness of Doughs

The ability to adhere to 45°-inclined plexiglass surfaces (50 mm internal diameter, 25 mm internal depth) under large deformations preceding and exceeding the fracture point was evaluated. Dough surface properties were determined by performing a loading–unloading test, using two complementary conical probes. The same amount of dough specimen was loaded in the receiving conical probe; then, it was left to rest for 10 min to fully recover all internal stresses. During the loading step, the specimen was compressed and the load-line displacement rate was set at 50 mm·min^−1^ (0.00083 m·s^−1^) up to a gap of 8 mm between the conical surfaces, allowing the dough to exceed its fracture point force. The unloading step was performed immediately after the loading step, allowing the tension load to be registered during full detaching of the specimen from the plexiglass surfaces.

The maximum compressive load measured during the loading step for the spreading resistance and the maximum tension load during the unloading step for stickiness were determined. The test was replicated 10 times for each formulation.

#### 2.3.3. Texture Profile Analysis of Biscuits

The load vs. time curves acquired in two loading–unloading cycles were used to stimulate the two bites. The parameters such as the springiness, hardness, cohesivity, friability, fragility, resiliency, and chewiness were determined. The test consisted of 0.5 N preload, loading the biscuit at 10 mm·min^−1^ to 25% deformation, unloading at 400 mm·min^−1^ and resting for 60 s for fast recovery, reloading the cookie at 10 mm·min^−1^ to 25% deformation, and unloading at 400 mm·min^−1^ and resting for 60 s for recovery.

The biscuits used for texture profile analysis were characterized by an average size of 50 × 30 × 12 mm for the width, length, and thickness, respectively. The test was repeated 10 times for each formulation.

#### 2.3.4. Fracture Test of Biscuits by Single-Edge Notched Three-Point Bending Test

The biscuit was analyzed in its original shape and geometry after making a notch with a depth of 2 mm at the center of the convex surface. The alignment of the notch on the biscuit surface with the loading tool was ensured and a non-destructive preload of 0.1 N was imposed. The fracture test was carried out with a load-line displacement speed of 1 mm·min^−1^ under position control conditions.

Load vs. time and load vs. load-line displacement data were numerically analyzed by preforming first, second, and third derivative analysis to calculate the Young’s elastic modulus (E), together with the work required to initiate and work required to propagate the fracture within the biscuit ligament. At least 10 replications were performed for each biscuit.

#### 2.3.5. Color Analysis of Biscuits

Baking performance was evaluated by determining changes in color attributes. A digital camera (Panasonic, DMC-FZ1000, Osaka, Japan) operating with high resolution (pixel size of 5.7 micron) was used under uniform light to acquire the images of the biscuit surfaces. The size calibration was performed using a phantom tool with standardized sizes.

L* (Lightness; values range from 0-black to 100-white), a* (positive values indicate the degree of redness, while negative values the degree of greenness), and b* (positive values indicate the degree of yellowness, while negative values the degree of blueness) of the CIE Lab system were considered and analyzed using Adobe Photoshop (2020, 21 x). The values of L*, a*, and b* were extracted by using the histogram function and were converted into the standard values, as reported by [28]. Then, chroma (C*), hue angle (hab), total color difference (ΔE), whiteness index (WI) and yellowness index (YI) were calculated according to CIE 1976 L*a*b* color space [29]. These indices were determined according to Pathare et al. [30].

### 2.4. Determination of Glucosinolates

Broccoli-derived ingredients (i.e., flour and hydroalcoholic extracts) plus the enriched biscuits were analyzed for their glucosinolate (GLS) content, subsequent to extraction according to [16] with minor modifications. BF (0.5 g) and biscuits (1 g) were subjected to extraction with 3 and 2 mL of methanol/water (70:30, *v*/*v*) at 70 °C for 30 min using a thermoblock (Falc Instruments srl, Model TA120P1, Bergamo, Italy) and then were sonicated for 15 min. The extraction and the analysis were performed in triplicate. The mixture was centrifuged (Remi Elektrotechnik Ltd., Neya 16R, Mumbai, India) at 4500 rpm for 5 min, and the supernatant was collected in a 2 mL Eppendorf tube. The supernatant was dried using an integrated speedvac system (Thermo Fisher Scientific, Model ISS110-230, Waltham, MA, USA) and the residue was dissolved in ultrapure water. The retrieved parts of BF and biscuits along with the extracts were filtered through a 0.45 µm filter (Sartorius Regenerated Cellulose membrane) and 2 µL was injected into a UPLC-PDA-MS system (Waters Corporation, Acquity, Massachusetts, USA). The separation was performed on a CSH C18 (100 × 2.1 mm, 1.7 µm) column according to Nartea et al. [31]. The mobile phase consisted of A (0.1% formic acid in water, *v*/*v*) and B (0.1% formic acid in acetonitrile, *v*/*v*). The gradient was as follows: 96% A and 4% B, 0 min; 85% A and 15% B, 10 min; 30% A and 70% B, 20 min; 30% A and 70% B, 25 min; 96% A and 4% B, 30 min. The flow rate was kept constant at 0.3 mL·min^−1^. The column temperature was set at 35 °C and sample loading was performed at 20 °C. PDA spectra were recorded from 200 to 500 nm. The MS was operated in negative electrospray ionization mode in full scan at a range of 50–600 *m*/*z*; the cone voltage was 15 V; and the capillary voltage was 0.8 kV. Retention time and mass spectra were used to identify and further quantify GLSs.

### 2.5. Total Phenolic Content

BF and the biscuits (1 g) were mixed with 15 mL of ethanol/water (50:50, *v*/*v*) at 40 °C for 1 h in the dark. Then, they were centrifuged, and the supernatant was collected. The total phenolic content (TPC) of BF, the extracts and the biscuits was determined according to the Folin–Ciocalteu method [32]. The mixture was composed of 20 µL of the sample, 1.58 mL of water, and 100 µL of Folin reagent. After 7 min, 300 µL of sodium carbonate solution was added to the mixture, vortexed and left at room temperature for 30 min. The absorbance was measured at 750 nm in a UV-Vis spectrophotometer (Onda, UV-31 SCAN, Beijing, China). Each sample was analyzed in triplicate and the results were expressed as mg gallic acid equivalents (GAE)·g^−1^ of the sample, using a calibration curve of gallic acid.

### 2.6. Simultaneous Determination of Carotenoids and Tocopherols

Carotenoids and tocopherols from BF and the biscuits were extracted according to [33] with slight modifications. Each sample (100 mg) was added to acetone (5 mL), kept for 15 min at 4 °C, vortexed, and centrifuged (Remi Elektrotechnik Ltd., Neya 16R, Mumbai, India) at 3000 rpm for 5 min at 4 °C. The extraction was repeated twice. The supernatants were collected, filtered (0.45 µm Regenerated Cellulose, Sartorius), dried and resuspended in 1 mL of acetone. Finally, 3 µL was injected into a UPLC-PDA-FLR system (Waters Corporation, Acquity, MA, USA). The column was a HSS T3 C18 (100 × 2.1 mm, 1.8 µm). The chromatographic conditions were set according to [31]. PDA was set at 450 nm to detect carotenoids, while for tocopherols, FLR was at 290 nm excitation energy and 330 nm emission energy. Carotenoids and tocopherols were identified via the comparison of retention time and absorbance spectra with pure standards. Their quantification was performed by means of external calibration. Each sample was analyzed in triplicate.

### 2.7. Statistical Analysis

Elaborated data were analyzed using STATISTICA v. 10 (StatSoft, Inc., Tibco, CA, USA). One-way ANOVA and Tukey tests were applied to identify statistical differences (*p* < 0.05) among samples in terms of bioactive compounds. Principal component analysis (PCA) was performed based on correlation matrix data, factor coordinates and eigenvalues analysis. To obtain insights on the interplay between the dough enrichment strategy and THE final quality of the enriched biscuits, the texture properties of the biscuits were treated as input active variables in PCA, while color properties were treated as supplementary variables.

## 3. Results

### 3.1. Rheological Properties of Doughs

**Figure 2:**
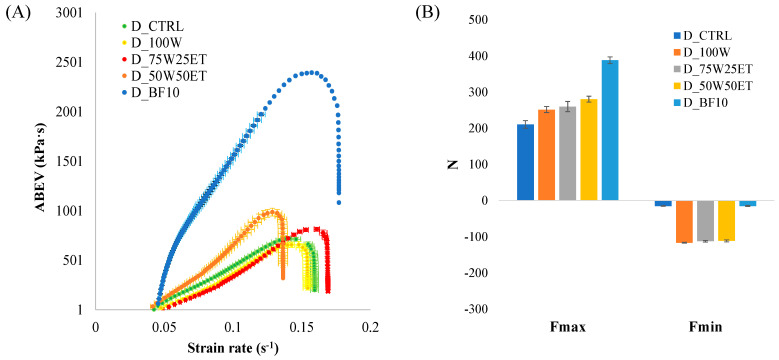
Rheological properties of doughs: (**A**) biaxial extension of the doughs under large strain lubricated compressive conditions; (**B**) spread and stickiness of the doughs under large-strain conditions. Error bars represent the confidence interval consisting of two times the standard errors around the mean values.

Figure 2a shows changes in the apparent extensional viscosity (kPa·s) as a function of the strain rate (s^−1^) in the dough.

As can be inferred from Figure 2A, a fracture point is reached during the lubricated biaxial compression test which corresponds to the maximum ABEV value. Significant differences are observed (*p* < 0.001) among the investigated dough formulation both in terms of maximum ABEV and maximum hardening rate, as evaluated within the full range of strain rate that precedes the fracture point of the dough structure (Table S1). The ABEV values follow the order 100W < STD < 75W25ET < 50W50ET < BF10, while the strain hardening rate follows the opposite order.

The partial substitution of sunflower oil with broccoli extracts led to significant changes in the extensional resistance and strain hardening rate compared to D_CTRL. Such a result suggested that the higher the level of ethanol used for the extraction, the higher the extraction of structural active compounds (e.g., polysaccharides, pectin, flavonoids, phenols) which were able to interact with wheat flour hydrocolloids under strain.

On the other hand, the partial substitution of wheat flour in the dough preparation with BF led to a dough (D_BF10) with the highest resistance to the biaxial extension, as suggested by the highest level of ABEV (2395.63 kPa∙s) and the lowest hardening strain rate (5.1 × 10^−5^ kPa∙s^2^), being 3.3 times higher and 0.3 times lower than D_CTRL, respectively. Such a result suggested that the broccoli’s hydrocolloids, mainly consisting of lignin, cellulose, hemicellulose, and pectin play a major role in dough consistency and workability with respect to the wheat flour hydrocolloids. Lignin, cellulose, and hemicellulose behave as elastic and viscoelastic structural elements in vegetable matrices such as cauliflower and broccoli, while pectin plays a plasticizing role [34].

The spread (maximum compressive load, F_max_) and the stickiness (maximum tension load, F_min_) of doughs are shown in Figure 2B.

F_max_ followed the order D_CTRL < D_100W < D_75W25ET < D_50W50ET < D_BF10. Such results were consistent with those related to the extensional resistance, except for the spread resistance of D_100W, which was significantly higher than D_CTRL, irrespective of the observed ABEV values. As far as the stickiness properties are concerned, it is worth noting that no significant difference between D_CTRL and D_BF10 was detected, while the doughs prepared with hydroalcoholic extracts were almost 7.7 times stickier than D_CTRL and D_BF10. This suggests the ability of hydro-alcoholic mixture to extract from broccoli a significant amount of relatively low-molecular-weight polysaccharides which are able to modify the surface properties under strain of the enriched doughs. Consequently, our doughs were limited to 30 mL of hydroalcoholic extracts to allow dough workability and to avoid more stickiness.

Based on the bulk and surface properties, as expressed in terms of apparent extensional viscosity, strain hardening rate, maximum load to spread and stickiness, as well as on the practice used to prepare the investigated doughs, we considered D_BF10 as the functional dough with the best workability features and the highest amount of bioactives from broccoli.

### 3.2. Rheological Properties of Biscuits

**Figure 3:**
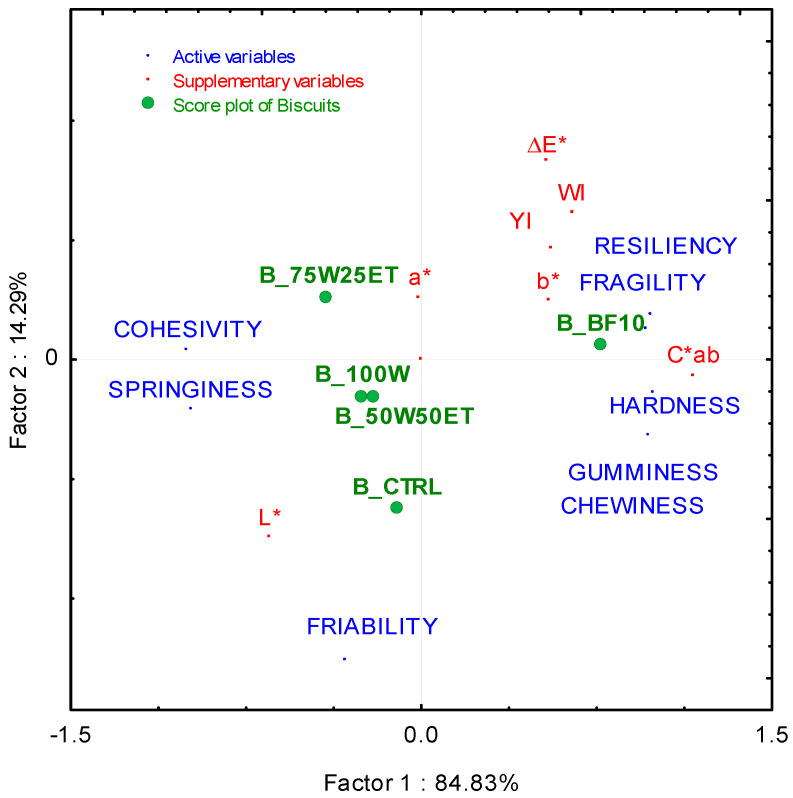
PCA plot score using the texture (active variables, blue) and color (supplementary variables, red) attributes of biscuits (green). The supplementary variables are as follows: L*, lightness; a*, redness; b*, yellowness; YI, yellowness index; WI, whiteness index; ΔE*, total color difference; C*ab, chroma.

Texture properties of biscuits (Table S2) were used as active variables to perform PCA, while color attributes (Figure S1) were used as supplementary variables. The conducted PCA displayed 99.12% of the total variance, with PC1 explaining the most (84.83%) (Figure 3).

B_BF10 appeared to be the hardest, most fragile, and most resilient biscuit and, at the same time, was the least springy and friable among all the enriched products. B_75W25ET showed the opposite textural features, while B_CTRL, B_100W, and B_50W50ET displayed an intermediate behavior. In detail, B_BF10 showed the lowest springiness (related to the extent of recovery of the original shape from the compressed state) and the highest resiliency (rate of recovery of the original shape after compression), while no differences were detected among the other biscuits, including the control. Such results suggest that BF provided a significant quantity of hydrocolloids, resulting in the highest elastic and viscoelastic interaction in the final biscuit. Moreover, these interactions enable air volume entrapment during cooking, and therefore B_BF10 also displayed the greatest work to initiate and work to propagate a fracture (Figure S2 and Table S3).

According to the derived texture properties, B_75W25ET showed the lowest chewiness, more likely due to the highest quantity of compounds having plasticizing effects on the biscuit structure strain and fracture. In contrast, the chewiness of B_BF10 was the highest among the investigated biscuits. Meanwhile, in gluten-free products, Krupa-Kozak et al. [24] highlighted an increase in chewiness of bread prepared with 5% broccoli leaf powder. The highest chewiness and hardness registered for B_BF10 suggested that the biscuit requires a longer time for oral processing and a higher number of chews before swallowing [35]. It has been reported that a higher number of masticatory cycles increases satiety and reduces food intake, which could be helpful for targeting eating disorders [36].

Regarding color attributes, B_BF10 showed great differences compared to the control and the other enriched biscuits in terms of colorfulness (ΔE), WI, YI, C*, and b* parameters. This could be attributed to the abundance of colored components such as phenols and carotenoids. Color is important to meet consumer acceptability [25]. In a previous study, the incorporation of broccoli by-product in crackers was positively evaluated [10]. Therefore, the color of B_BF10 could be appreciated by consumers.

### 3.3. Glucosinolate Content in Broccoli, Extracts, and Biscuits

Nine GLSs were identified in BF, among which glucoraphanin, glucobrassicin, and neoglucobrassicin were the most abundant (421.4 ± 19.7, 369.7 ± 7.9, and 283.9 ± 7.3 µg·g^−1^ DW, respectively) (Table 2).

These GLSs are predominant in broccoli florets, as reported by Li et al. [16], who characterized GLSs in different organs of 80 broccoli genotypes. Although GLSs are hydrophilic compounds, none were detected in the extract obtained with 100% water (E_100W). As reported by Bojorquez-Rodríguez et al. [27], desulfated GLSs in the water extracts were limited to certain ones, namely glucoiberin, progoitrin, 4-hydroxyglucobrassicin, 1-hydroxy-3-indoylmethyl, with low concentrations (1.3, 0.3, 0.4, and 0.3 g·kg^−1^ DW, respectively).

In the other hydro-alcoholic extracts, the higher the amount of ethanol, the higher the concentration of GLSs recorded, for a total of 9749.1 ± 15.3 µg·g^−1^ DW in E_50W50ET. A similar trend was observed by Bojorquez-Rodríguez et al. [27] in broccoli sprouts, where the concentration of GLSs in the extracts with 50% ethanol was much higher than that with 0% ethanol. The total GLS concentrations detected in E_50W50ET was almost 8 times higher than in BF. This could be attributed to the extraction method, and especially to the sample/solvent ratio used (1:15 in _50W50ET vs. 1:6 in BF). Pham et al. [37] noticed that a high sample-to-solvent ratio limited the dissolution of compounds into the solvent due to a saturation effect.

Unfortunately, no GLSs were detected in biscuits enriched with hydroalcoholic extracts or that made with E_50W50ET, despite the high GLS concentration. This could be related to the limited amount of extract which could be added into the biscuit formulation (see Section 3.1). Therefore, considering the final concentration of GLSs achieved in the extracts accompanied by the limitation of the stickiness of the dough, hydroalcoholic extracts are not suitable ingredients to significantly fortify bakery goods. However, they could be valuable ingredients for other liquid or semi-solid formulations by providing a considerable amount of health-promoting GLSs.

On the other hand, seven out of nine GLSs were identified in the biscuit enriched with 10% BF for a total of 33.2 ± 3.4 µg·g^−1^ DW (Table 3).

Glucoraphanin accounted for 54.5% of the total. Glucoraphanin is particularly important because it can be degraded by enzymes of the intestine into sulforaphane, which is a strong chemo-preventive isothiocyanate [8,13,38,39,40]. For instance, Mirmiran et al. [41] noticed a significant reduction in the level of inflammatory markers after 4 weeks of supplementation with broccoli sprout powder with a high sulforaphane concentration.

### 3.4. Total Phenolic Content

**Figure 4:**
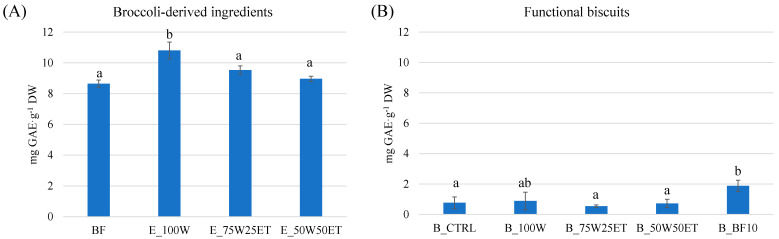
Total phenolic content in broccoli-derived ingredients (**A**) and enriched biscuits (**B**). Letters indicate statistical differences (*p* < 0.05).

The TPC of broccoli-derived ingredients varied from 8.7 to 10.8 mg GAE·g^−1^ DW, with E_100W displaying the highest value (Figure 4A).

Our findings are slightly higher compared to those reported by [17], who found a TPC of about 2.5 mg GAE·g^−1^ DW in florets. This could be related to the shorter extraction time these authors used (10 min vs. 1 h) or to the variety and other environmental factors, which could imply variation in the content of antioxidants [42].

Once the hydroalcoholic extracts were included in the biscuit formulation, they did not lead to a significant increase in TPC compared to the control biscuit (~0.73 mg GAE·g^−1^ DW; Figure 4B). By contrast, the TPC of B_BF10 was almost double this value (1.9 mg GAE·g^−1^ DW). Such a result suggests that the food matrix (BF) protects these compounds from thermal degradation [43]. A similar concentration was observed in crackers enriched with 15% broccoli stem flour [10]. The encapsulation of phenolic compounds is one of the most commonly used techniques to avoid their loss [22]. In this case, the incorporation of BF represents a cost-effective alternative to fortify biscuits with phenolic compounds.

### 3.5. Tocopherols and Carotenoids

**Figure 5:**
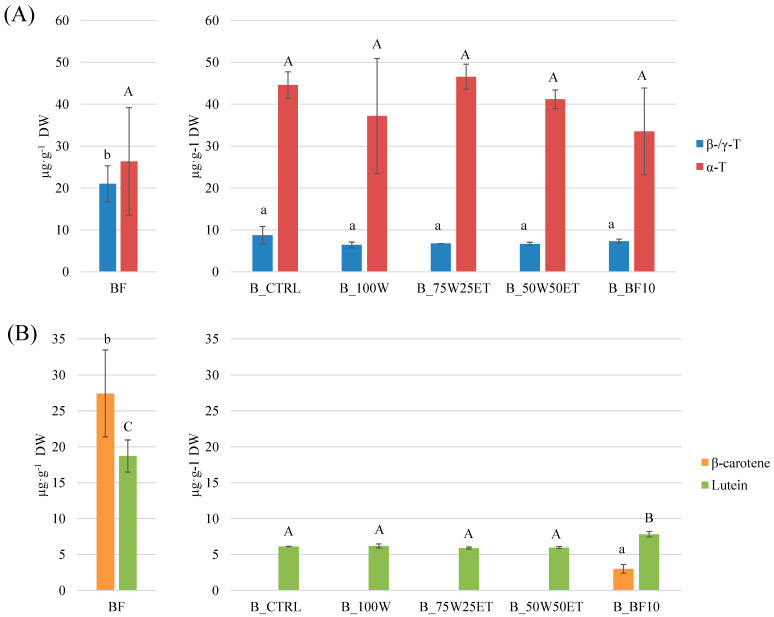
(**A**) Tocopherol and (**B**) carotenoid content in broccoli flour (BF) and biscuits. The letters indicate statistical differences (*p* < 0.05).

Among the broccoli-derived ingredients investigated, tocopherols and carotenoids were determined only in BF. Due to the lipophilic nature of these compounds, none of them were detected in the hydroalcoholic extracts. BF was mainly characterized by β/γ and α tocopherols (~47.4 µg·g^−1^ DW in total, Figure 5A).

Lee et al. [3] found concentrations ranging from 1.8 to 286 µg·g^−1^ DW of total tocopherols among twelve *Brassicaceae*. However, tocopherols are differently accumulated in different broccoli organs, with leaves having the highest concentration compared to florets and stems [17]. No statistical differences were found among enriched biscuits compared to the B_CTRL. However, the results from tocopherols analysis were characterized by a quite high data dispersion. This may be the result of the diverse composition of functional ingredients in low-molecular-weight compounds (e.g., polysaccharides, peptides) which affected the rheological and mechanical properties of doughs and biscuits, probably causing an ununiform oxidative deterioration of tocopherols during biscuit preparation and baking. It is noteworthy that the tocopherol fraction in B_CTRL can mainly be attributed to sunflower oil (used as an ingredient), which was reported to have a concentration of γ- and α- tocopherol of about 92 and 432 mg·kg^−1^ [44]. The sunflower oil was reduced by the hydroalcoholic extracts in the case of the enriched biscuits.

Regarding carotenoids, lutein and β- carotene were predominant in BF (27.4 and 18.7 µg·g^−1^ DW, respectively; Figure 5B). Previously, violaxanthin and neoxanthin were also identified in broccoli florets, with concentrations of about 30 µg·g^−1^ DW, such as β-carotene [3,17]. Different extraction methods might be more successful in obtaining a better carotenoids profile. The hydroalcoholic extracts were devoid of a carotenoid fraction. Hence, the biscuits made using them were not different from B_CTRL. The presence of lutein in B_CTRL and in the other biscuits containing hydroalcoholic extracts (approximately 6 µg·g^−1^ DW) can be attributed to wheat flour [45]. On the other hand, a 10% substitution level of BF led to a significant enrichment of B_BF10; namely, the content of β-carotene was 3.0 ± 0.6 µg·g^−1^ DW and that of lutein was 7.8 ± 0.4 µg·g^−1^ DW. The recovery rate was 166% and 137% for β-carotene and lutein, respectively. Potentially, this confirms that there is no effect of the thermal treatment on degrading these compounds, and in actual fact it promoted their release from the matrix. Previously, [31] enriched pizzas with 10 and 30% cauliflower flour, obtaining a proportional increase in the carotenoid content. However, the recovery rates in pizzas enriched at 10% ranged between 12 and 57%. The lower recovery rates registered could mostly be related to yeast fermentation. Hence, B_BF10 could be an alternative source of vitamin A which could be introduced in a balanced daily diet.

## 4. Conclusions

The transformation of broccoli by-products into functional ingredients, such as hydroalcoholic extracts and flour, for food applications represents a good circular economy approach and a cost-effective alternative to valorize them. The substitution of sunflower oil with hydroalcoholic extracts in biscuit formulations was not suitable to guarantee proper enrichment because of the enhanced stickiness and poor workability of the doughs observed. However, the high glucosinolate content suggests them as potential ingredients for liquid or semi-solid applications.

On the other hand, the incorporation of 10% broccoli flour resulted in a cohesive and easy-to-work dough with enhanced nutritional quality in terms of the glucosinolate, carotenoid and phenolic content. The matrix helped to prevent the thermal degradation of these sensitive compounds. Therefore, broccoli flour is a promising functional ingredient to fortify biscuits, and potentially other bakery goods. Biscuits with broccoli flour will be further analyzed to evaluate their shelf-life as well as consumer acceptance through sensory analysis. The future perspective involves developing these functional biscuits at the industrial scale.

## Figures and Tables

**Table 1:** List of ingredients in control and enriched biscuits.

	B_CTRL	B_BF10	B_100W	B_75W25ET	B_50W50ET
Flour “00” (g)	500	430	500	500	500
BF (g)	0	70	0	0	0
Cream of tartar (g)	8	8	8	8	8
Sucrose (g)	200	200	200	200	200
Sodium bicarbonate (g)	2.5	2.5	2.5	2.5	2.5
Sodium chloride (g)	1.5	1.5	1.5	1.5	1.5
Egg (g)	162	169	164	169	156
Milk (mL)	10	10	10	10	10
High oleic sunflower oil (mL)	140	140	110	110	110
Extract 100W (mL)	0	0	30	0	0
Extract 75W25ET (mL)	0	0	0	30	0
Extract 50W50ET (mL)	0	0	0	0	30

B_CTRL: control biscuit; B_BF10: biscuit with 10% broccoli flour; B_100W: biscuit with 100:0 water/ethanol extract; B_75W25ET: biscuit with 75:25 water/ethanol extract; B_50W50ET: biscuit with 50:50 water/ethanol extract.

**Table 2:** Concentration of glucosinolates in broccoli-derived ingredients (flour and liquid extracts). Results are expressed as µg·g^−1^ DW. Values are reported as average ± standard deviation.

	BF	E_100W	E_75W25ET	E_50W50ET
Glucoraphanin	421.4 ± 19.7	Nd	668.6 ± 3.6	7107.8 ± 8.9
Glucoalyssin	12.5 ± 0.7	Nd	Nd	Nd
Gluconapin	2.8 ± 0.6	Nd	Nd	Nd
4-Hydroxyglucobrassicin	12.0 ± 0.4	Nd	Nd	Nd
Glucoerucin	1.3 ± 0.9	Nd	Nd	22.6 ± 0.5
Glucobrassicin	369.7 ± 7.9	Nd	Nd	1312.7 ± 5.5
Gluconasturtiin	41.1 ± 3.1	Nd	Nd	121.7 ± 1.0
4-Methoxyglucobrassicin	58.1 ± 2.1	Nd	280.7 ± 1.1	286.1 ± 1.2
Neoglucobrassicin	283.9 ± 7.3	Nd	Nd	898.3 ± 3.9
Total	1222.7 ± 50.9	Nd	949.3 ± 2.3	9749.1 ± 15.3

Nd: not detected; BF: broccoli flour; E: extract; W: water; ET: ethanol; CTRL: control; BF10: 10% broccoli flour.

**Table 3:** Concentration of GLSs in the functional biscuit made with 10% broccoli flour. Results are expressed as µg·g^−1^ DW. Values are reported as average ± standard deviation.

	B_BF10
Glucoraphanin	18.1 ± 1.6
Gluconapin	0.7 ± 0.4
4-Hydroxyglucobrassicin	0.6 ± 0.2
Glucobrassicin	5.7 ± 1.1
Gluconasturtiin	0.5 ± 0.1
4-Methoxyglucobrassicin	2.0 ± 0.4
Neoglucobrassicin	3.9 ± 0.4
Total	33.2 ± 3.4

## Data Availability

The data supporting the findings of this study will be available upon request.

## Supplementary Materials

The following supporting information can be downloaded at: https://www.mdpi.com/article/10.3390/antiox12122115/s1, Figure S1: Color attributes of control and functional biscuits prepared with broccoli-derived ingredients. Values are reported as average ± standard error. The color attributes reported are as follows: hab, hue angle; C*ab, chroma; YI, yellowness index; WI, whiteness index; ΔE*, total color difference; Figure S2: Work to initiate and propagate a fracture in cookies under plain-strain conditions; Table S1: Hardening rate and apparent biaxial extensional viscosity (ABEV) values of control and functional; Table S2: Texture profile analysis. Values are reported as average ± standard error; Table S3: Work to initiate and work to propagate a fracture of control and functional biscuits.
