# Supplementary material for: Bioactives and Technological Quality of Functional Biscuits Containing Flour and Liquid Extracts from Broccoli By-Products
Source: Antioxidants (Basel). 2023 Dec 14;12(12):2115. doi: 10.3390/antiox12122115 (PMC10740713; doi:10.3390/antiox12122115)
Supplement: Supplementary file 1 [file antioxidants-12-02115-s001.zip › antioxidants-2744316-supplementary.pdf]

Table S1. Hardening rate and apparent biaxial extensional viscosity (ABEV) values of control and functional doughs.

	**Hardening Rate** (kPa·s^2)	**Max ABEV** (kPa·s)
D_CTRL	1.7 E-04^{a}	721.28^{A}
D_100W	1.8 E-04^{b}	657.86^{B}
D_75W25ET	1.7 E-04^{a}	813.70^{C}
D_50W50ET	1.1 E-04^{c}	987.43^{D}
D_BF10	5.1 E-05^{d}	2395.63^{E}

D: dough; CTRL: control; W: water; ET: ethanol; BF10: 10% broccoli flour. Values represent average values. Different letters indicate significant differences with $p< 0.05$.

Table S2. Texture profile analysis. Values are reported as average ± standard error.

	**B_CTRL**	**B_100W**	**B_75W25ET**	**B_50W50ET**	**B_BF10**
Cohesivity [A2/A1]	3.58 ± 0.18	3.63 ± 0.15	3.69 ± 0.21	3.59 ± 0.17	3.22 ± 0.41
Springiness [D2/D1]	2.00 ± 0.02	2.01 ± 0.00	2.01 ± 0.00	2.00 ± 0.00	1.81 ± 0.06
Gumminess [HxC]	1192 ± 85.7	1083 ± 52.1	885 ± 85.5	1107 ± 98.6	1604 ± 90.1
Chewiness [HxCxS]	2385 ± 173	2180 ± 106	1781 ± 172	2216 ± 197	2941 ± 250
Resiliency [A4/A3]	0.07 ± 0.01	0.07 ± 0.00	0.06 ± 0.01	0.07 ± 0.01	0.15 ± 0.00
Fragility [1st peak]	0.06 ± 0.01	0.05 ± 0.01	0.05 ± 0.01	0.05 ± 0.01	0.1 ± 0.03
Friability[all peaks]	197 ± 3.33	190 ± 6.60	178 ± 4.85	190 ± 3.31	180 ± 2.86
Hardness[max peak]	333 ± 18.9	303 ± 19.2	242 ± 23.5	308 ± 20.8	539 ± 42.7

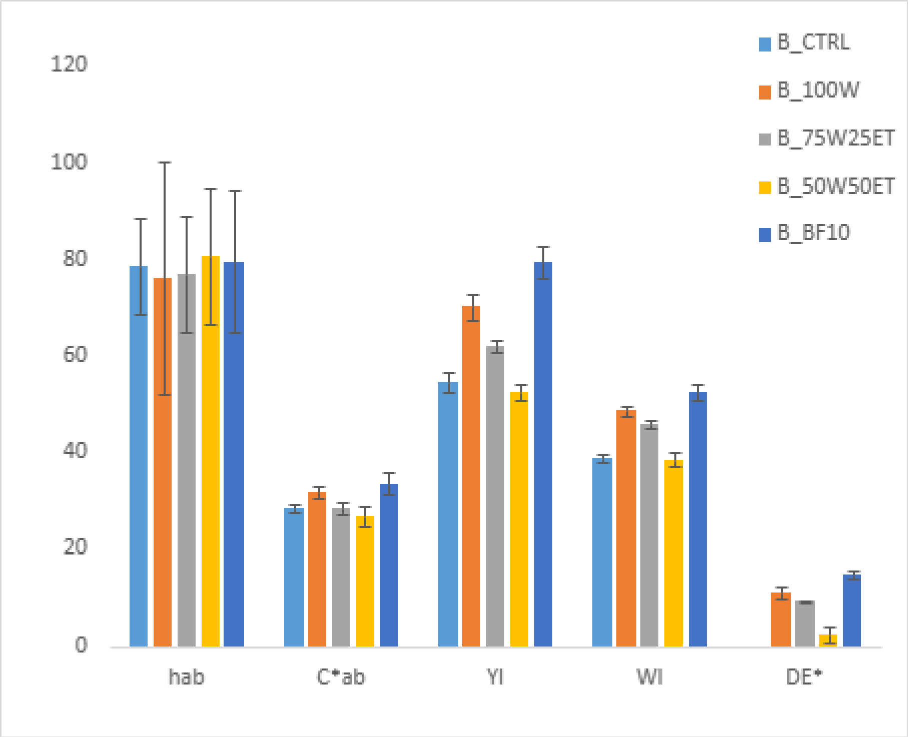

Figure S 1. Color attributes of control and functional biscuits prepared with broccoli-derived ingredients. Values are reported as average ± standard error. The color attributes reported are: hab, hue angle; C*ab, chroma; YI, yellowness index; WI, whiteness index; ΔE*, total color difference.

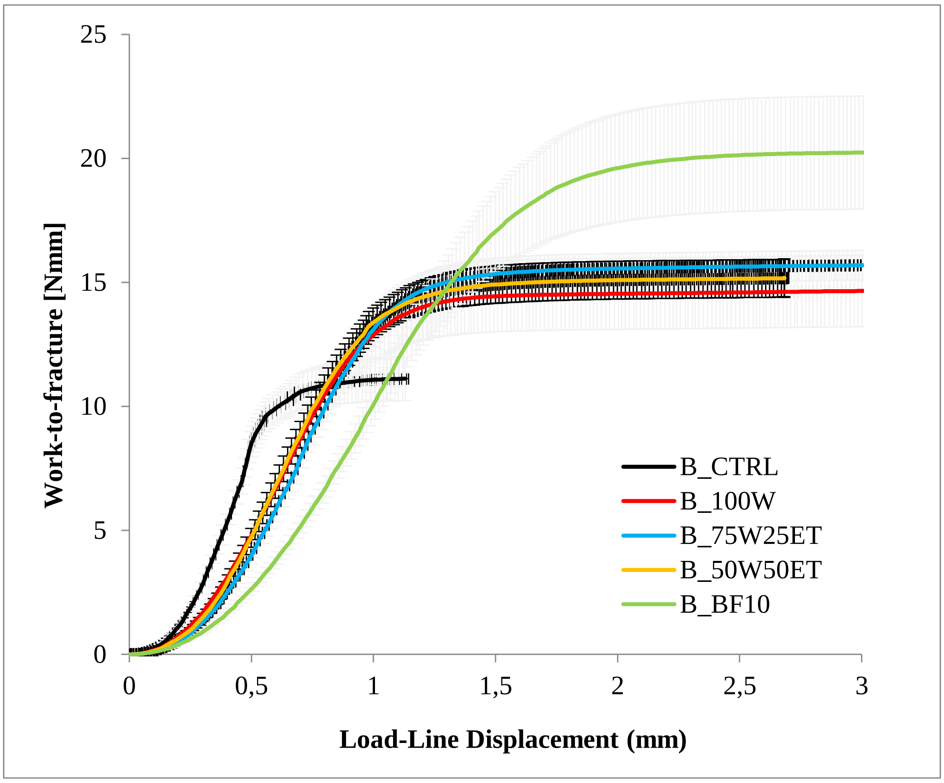

Figure S 2. Work to initiate and propagate a fracture in cookies under plain-strain conditions

Table S3. Work-to-initiate and work-to-propagate a fracture of control and functional biscuits.

	**Work-to-Initiate** (Nmm)	**Work-to-Propagate** (Nmm)
B_CTRL	7.03[a]	0.100[a]
B_100W	8.07[c]	0.110[b]
B_75W25ET	7.85[b]	0.121[c]
B_50W50ET	8.38[d]	0.131[d]
B_BF10	12.15[e]	0.650[e]

B: biscuit; CTRL: control; W: water; ET: ethanol; BF10: 10 % broccoli flour. Values represent average values. Different letters indicate significant differences with $p < 0.05$.
